# Red Propolis as a Source of Antimicrobial Phytochemicals: Extraction Using High-Performance Alternative Solvents

**DOI:** 10.3389/fmicb.2021.659911

**Published:** 2021-06-08

**Authors:** Cíntia M. dos Santos, Leonardo M. de Souza Mesquita, Anna Rafaela C. Braga, Veridiana V. de Rosso

**Affiliations:** ^1^Postgraduate Program in Nutrition, Universidade Federal de São Paulo (UNIFESP), São Paulo, Brazil; ^2^Postgraduate Program in Interdisciplinary Health Science, Universidade Federal de São Paulo (UNIFESP), São Paulo, Brazil; ^3^Department of Chemical Engineering, Universidade Federal de São Paulo (UNIFESP), São Paulo, Brazil; ^4^Nutrition and Food Service Research Center, Universidade Federal de São Paulo (UNIFESP), São Paulo, Brazil

**Keywords:** Brazilian propolis, red propolis, flavonoids, ionic liquids, eutectic solvents

## Abstract

Propolis is a resinous material rich in flavonoids and involved in several biological activities such as antimicrobial, fungicide, and antiparasitic functions. Conventionally, ethanolic solutions are used to obtain propolis phytochemicals, which restrict their use in some cultures. Given this, we developed an alcohol-free high-performance extractive approach to recover antibacterial and antioxidants phytochemicals from red propolis. Thus, aqueous-solutions of ionic liquids (IL) and eutectic solvents were used and then tested for their total flavonoids, antioxidant, and antimicrobial activities. The surface-responsive technique was applied regarding some variables, namely, the time of extraction, the number of extractions, and cavitation power (W), to optimize the process (in terms of higher yields of flavonoids and better antioxidant activity). After that, four extractions with the same biomass (repetitions) using 1-hexyl-3-methylimidazolium chloride [C_6_mim]Cl, under the operational conditions fixed at 3.3 min and 300 W, were able to recover 394.39 ± 36.30 mg RuE. g^−1^ of total flavonoids, with total antioxidant capacity evaluated up to 7595.77 ± 5.48 μmol TE. g^−1^_dried biomass_, besides inhibiting the growth of *Staphylococcus aureus* and *Salmonella enteritidis* bacteria (inhibition halo of 23.0 ± 1.0 and 15.7 ± 2.1, respectively). Aiming at the development of new technologies, the antimicrobial effect also presented by [C_6_mim]Cl may be appealing, and future studies are required to understand possible synergistic actions with propolis phytochemicals. Thereby, we successfully applied a completely alcohol-free method to obtain antimicrobials phytochemicals and highly antioxidants from red propolis, representing an optimized process to replace the conventional extracts produced until now.

## Introduction

Propolis is associated broad spectrum of activities, namely, fungicide (Marques das Neves et al., 2016), antioxidant, and antiparasitic (do Nascimento et al., 2016; Dantas Silva et al., 2017). Moreover, the impact generated by the pandemic COVID-19 has motivated the studies of propolis in action against infection by SARS-CoV-2 (Berretta et al., 2020; Refaat et al., 2021), whereas this natural product has anti-inflammatory properties (Lima et al., 2014; Washio et al., 2015; Kitamura et al., 2018) and action against some virus species (Hazem et al., 2017; Kwon et al., 2020). Recently, a clinical trial conducted in Brazil (National Clinical Trial Number: NCT04480593) has shown the efficacy of green propolis in supporting treatment in patients hospitalized with COVID-19 (Silveira et al., 2021).

Brazilian red propolis demonstrated strong cytotoxic potential *in vitro*, suggesting a potential therapeutic alternative for treatment against Chagas disease (Dantas Silva et al., 2017), schistosomiasis (Silva et al., 2021), and some types of cancer (da Silva Frozza et al., 2013; Dantas Silva et al., 2017; Banzato et al., 2020). These properties are related to propolis complex chemical composition, including formononetin, isoliquiritigenin, liquiritigenin, and biochanin A, as described by da Silva Frozza et al. (2013).

Traditionally, ethanolic solutions are the most conventional solvent used to obtain phytochemicals from red propolis. However, hydroalcoholic extracts’ consumption has some restrictions in specific cultures (Muslim; Al-Ansari et al., 2019). Also, envisioning the purification of some specific molecules with different polarities, other volatile organic solvents (VOSs) are used such as hexane, methanol, and chloroform (Oldoni et al., 2011; Marques das Neves et al., 2016). These solvents are widely used in several industrial segments; however, beyond toxicity, there are risks for workers (high-volatility) and damage to the environment (environmental toxicity). Thus, alternative technologies have been required in order to replace these solvents with more sustainable alternatives (Choi and Verpoorte, 2019). In this scenario, the development of extraction methods using alternative solvents to replace VOS becomes a promising strategy for future commercial applications, which increases the number of applications in some industrial sectors, such as food, cosmetic, and pharmaceutic ones. Among the alternative solvents, ionic liquids (ILs) and eutectic solvents (ESs) stand out as promising candidates to replace VOS (Passos et al., 2014; Ventura et al., 2017). The non-volatile and flammability suggest that these compounds are an essential alternative in developing new extractive processes (Nam et al., 2015). Ionic liquid, for example, can assume several properties, it has been applied to obtain several molecules with different polarities, such as carotenoids (hydrophobic compounds; Xiao et al., 2018; de Souza Mesquita et al., 2019, 2020a,b; Murador et al., 2019a,b), as well as flavanones (hydrophilic compounds), depending on the ionic composition (Xiao et al., 2018). Another advantage of IL is the possibility to recycle and reuse, thus contributing to a low carbon footprint in the process (de Souza Mesquita et al., 2019, 2020a,b). The ES also has a high potential to extract bioactive compounds from natural products, including flavonoids and phenolic compounds, since they are considered as designer solvents and IL. In some cases, ES could improve the bioavailability and antioxidant activity of the extracts, which are considered as a promising characteristic for new applications, especially in the food sector (Meng et al., 2018; Funari et al., 2019; Liu et al., 2019; Murador et al., 2019a).

Using an alternative extraction strategy in terms of high-performance and transposing the limitations on the use of ethanol would make it possible to obtain a commercially differentiated red propolis extract obtained by alternative solvents. Thereby, this work aims to establish a new method of extracting antioxidant and antimicrobial phytochemicals from red propolis using alternative solvents, creating new possibilities for applying this biomass, which is considered as extremely important for different purposes.

## Material and Methods

### Materials

The following standards were used: rutin hydrate, formononetin, daidzein, and 6-hydroxy-2,5,7,8-tetramethylchroman-2-carboxylic acid (Trolox) from Sigma-Aldrich (Darmstadt, Germany). The reagents 1-methylimidazolium, 1-chlorobutane, and potassium hexafluorophosphate; IL 1-n-butyl-3-methylimidazolium tetrafluoroborate ([C_4_mim][BF_4_]) and 1-hexyl-3-methylimidazolium chloride ([C_6_mim]Cl); choline chloride, glycerol, 1,4-butanediol, and levulinic acid from Sigma-Aldrich (Darmstadt, Germany). Reagents sodium nitrite (Vetec®) and aluminum chloride (Proquímicos®); Fluorescein sodium salt and α, α′-Azodiisobutyramidine dihydrochloride (AAPH) from Sigma-Aldrich (Darmstadt, Germany). Culture mediums: Mueller Hinton Agar (Himedia®) and Nutrient broth (Himedia®). *Salmonella enteritidis* ATCC 13076 and *Staphylococcus aureus* ATCC 19095 were supplied by the Bacterial Culture Collection of the Oswaldo Cruz Institute – FIOCRUZ (Manguinhos, Rio de Janeiro, Brazil). Vancomycin (30 μg/disc) and Meropenem (10 μg/disc; DME®) antibiotic filter paper discs and inert filter paper discs (DME®).

### Samples

The samples of red propolis were purchased from an apiary located in Porto de Pedras’ municipality, in the state of Alagoas (Latitude: 9° 09' 11" S, Longitude: 35° 17'19" 17'19" W) in April 2018, by donation. The sample was composed of a single batch consisting of 350 g of sample and was lyophilized for 48 h, processed using an analytical mill (Ika®, model A11 Basic), vacuum-packed in sealer (TECMAQ®, model TM250), and maintained frozen at −40°C.

### Extraction of Flavonoids From Red Propolis

#### Convectional Extraction

Ethanol was applied as a control to obtain red propolis extract. Thus, results using ethanolic solutions (70 and 95% v:v) were compared and evaluated with the developed method’s extractive performance based on alternative solvents. The homogenization was performed in an ultrasonic probe (500 W, 20 kHz, 4 mm in diameter; Unique, model DES500, Brazil) at 400 W for 5 min, followed by centrifugation at 4700 rpm for 15 min (process repeated twice). The solid-liquid ratio (R_S/L_) was fixed at 1:3 (0.5 g of sample for 1.5 g of solvent). The procedure was performed in triplicate with three extractions for each sample. The supernatants were stored in a freezer at −40°C for further analysis.

#### Extraction of Flavonoids From Red Propolis With Ionic Liquids and Eutectic Solvents

##### Preliminary Tests

In total, four different IL were evaluated: 1-butyl-3-methylimidazolium tetrafluoroborate ([C_4_mim][BF_4_]) and 1-hexyl-3-methylimidazolium chloride ([C_6_mim]Cl) – which were obtained commercially, 1-butyl-3-methylimidazolium chloride ([C_4_mim]Cl) and 1-butyl-3-methylimidazolium hexafluorophosphate ([C_4_mim][PF_6_]) both synthesized in the laboratory, as described by Martins and de Rosso (2016). ([C_4_mim][BF_4_]) was synthesized in a round-bottom flask by mixing 0.10 mol of 1-methylimidazole and 0.10 mol of 1-chloro-butane. The mixture was stirred and refluxed (70–80°C) for 72 h. A viscous yellow liquid, which was washed twice with dichloromethane, was obtained. [C_4_mim]Cl was dried under vacuum at 100°C and crystallized at −40°C. [C_4_mim][PF_6_] was synthesized by a mixture containing 0.01 mol of [C_4_mim]Cl, and 0.01 mol of potassium hexafluorophosphate in distilled water was stirred vigorously for 45 min. The upper aqueous phase formed was separated and discarded; the remaining liquid was added to distilled water and stirred for 15 min. Then, 40 ml of chloroform was added. The solvent was evaporated under vacuum to yield a viscous liquid with a slightly yellow color. Besides, three ES were synthesized, associating choline chloride (CH) to three hydrogen donors: glycerol (CH–GLY), 1,4-butanediol (CH–BUT), and levulinic acid (CH–LEV), in a molar ratio of 1:2, as described by Liu et al. (2019). For each ES, 10% (g/g) water (co-solvent) was added and homogenized at 60°C for 10 min in a round-bottom flask.

The extractions were performed using a 1:3 solid-liquid ratio (*R*_S/L_; g/ml), as in conventional extraction. The samples were maintained in an ice bath, and the homogenization was performed in a 500 W, 4 mm in diameter, and 20 kHz ultrasonic probe (Unique, model DES500, Brazil) at 400 W for 5 min. Then, the samples were centrifuged at 4700 rpm for 15 min. This procedure was performed in triplicate with three extractions for each sample (using the same biomass). The supernatants were stored in a freezer at −40°C for further analysis.

##### Optimization of Extraction Conditions Using Response Surface Methodology

The most effective solvent for the extraction of flavonoids was submitted to a Central Composite Design (CCD) to optimize the process. The effects of the variables number of extractions, time of extraction (*T*_min_), and cavitation power (W) were assessed using the Central Composite Design Rotational (CCDR; 2^3^), with six axial points (calculated by the interpolation considering the experimental values of factorial and central points) and three central points, totaling 17 trials. ILs, as well as ES, it has a high viscosity, and since propolis biomass is a very resinous substance, both factors difficult the extraction process; Thus, the variable (*R*_S/L_) was fixed at 1:3, as it was the minimum amount of solvent that enabled a less viscous liquid for cavitation. The response was measured by total flavonoid yield and antioxidant activity using the oxygen radical absorbance capacity (ORAC) method.

##### Model Validation

An experiment contemplating the best extraction conditions was carried out, in triplicate, to validate the obtained model and verify the reproducibility of the method and compare the average of the extracts with the predicted model obtained in the experimental design.

### Quantification of Total Flavonoids

The total content of flavonoids present in red propolis extract was quantified according to the method exposed by Zhishen et al., 1999 colorimetric method, based on the reaction with aluminum chloride. The absorbance of the solution was determined at 510 nm in a Varian® spectrophotometer. Total flavonoid contents were calculated as mg of rutin equivalent (RuE). g^−1^ using a calibration curve ranging between 200 and 700 ppm (*R*^2^ = 0.998).

### Analysis of Flavonoids in High Performance Liquid Chromatography

The flavonoids were separated and chromatographically identified by HPLC-DAD Shimadzu (Kyoto, Japan; Atlantis® C-18 column, size 4.6 mm × 250 mm) with quaternary pump (model LC-20AT), a DAD detector (Shimadzu, model SPD-M20A), a degassing unit (DGU-20A5), and a Rheodyne injection valve with a 20 μl loop. The extracts were filtered through a 0.22 μl filter (Millipore). The column was eluted using a linear gradient of acidified water (solvent A) and methanol (solvent B), starting with 80% solvent B up to 10 min, gradually reducing solvent B by 10% over time 15, 30, 45, 60, and 65 min, maintaining only solvent A, between 65 and 70 min, and increasing to 80% solvent B until 90 min (pump flow: 0.800 ml/min). The UV-visible spectra were obtained between 200 and 600 nm, and the chromatograms were processed at 280 and 355 nm. The red propolis flavonoids were tentatively identified based on the following information: elution order, retention time, and UV-visible spectral features compared to those of the standards analyzed under the same conditions and the available literature data. Co-chromatography was carried out with formononetin and daidzein analytical standards.

### Evaluation to Deactivate Peroxyl Radicals by Hydrophilic Compounds in a Homogeneous System: Oxygen Radical Absorbance Capacity

The antioxidant activity against the peroxyl radical (ROO•) was measured by ORAC test, which is based on monitoring the effect of the hydrophilic or standard extract (Trolox) in the analysis of fluorescence resulting from the oxidation of fluorescein induced by ROO• radical, generated by decomposition of the 2,2′-azobis (2-methylpropionamidine) dihydrochloride (AAPH) at 37°C (Rodrigues et al., 2012).

The test was performed in 96-well microplates, where 150 μl of fluorescein (61 nm, prepared in 75 mm phosphate buffer pH 7.4) were transferred. Then, 25 μl of the propolis extracts were added in two different dilutions (1:1,000 and 1:10,000) in a phosphate buffer, or white (buffer solution only), or Trolox standard (control). The plate was incubated for 10 min at 37°C with intermittent shaking. After this time, 25 μl of AAPH solution (19 mm, prepared in phosphate buffer) was added to each well to generate radicals and microplate and inserted in the plate reader, where the fluorescence reading occurred at 538 nm after excitation at 485 nm, every minute, for 1 h and 30 min (Murador et al., 2016). The area under the fluorescence vs. time curve for the sample minus the area under the blank curve was compared with the standard Trolox curve. The results were expressed in μmol Trolox Equivalent (TE). g^−1^.

### Antimicrobial Activity Test

The disc diffusion technique was used to assess the propolis extracts’ antimicrobial activity, in which the extracts were applied to filter paper discs, and these, placed on Mueller Hinton agar plates, previously sown with bacterial suspensions. The Gram-positive bacteria *St. aureus* ATCC 19095 and Gram-negative bacteria *Sa. enteritidis* ATCC 13076 were used for antimicrobial tests (CLSI/NCCLS, 2003; Winn, 2008). *Salmonella enteritidis* ATCC 13076 and *St. aureus* ATCC 19095 were supplied by the Collection of Bacterial Cultures of the Oswaldo Cruz Institute – FIOCRUZ (Manguinhos, Rio de Janeiro, Brazil), where they were grown on nutrient agar and incubated at 37°C for 24 h, then lyophilized and stored in vacuum sealed ampoules at a temperature between −10 and −20°C and transported to our laboratory in a thermic box. Analysis of purity, viability, morphology, and identity was carried out on the used batch.

#### Preparation of Discs

Inert, sterile Petri dishes were placed inert filter paper discs (DME®), and these were soaked with 10 μl of the optimized extracts in the dilutions 25 mg ml^−1^, 75 mg ml^−1^ (in sterile saline), and pure extract. Inert discs were soaked with IL in the same conditions to characterize the negative control. For positive control, Vancomycin (30 μg/disc) and Meropenem (10 μg/disc; DME®) antibiotic filter paper discs were used for Gram-positive and Gram-negative bacteria, respectively.

#### Disc Diffusion Technique

The bacterial strains were reactivated, sowed in nutrient broth (tryptone soy broth), and incubated in a bacteriological oven at 36 ± 1°C for 24 h. Sample’s inoculum was prepared according to document M2-A8 of the National Committee of Clinical and Laboratory Standardization/Clinical and Laboratory Standards Institute (NCCLS/CLSI) by carrying out a direct suspension, in sterile saline, from the growth in broth. The suspension’s turbidity was adjusted by comparing it with the 0.5 tubes of the McFarland scale, that is, an inoculum containing approximately 1.0 × 10^8^ CFU/ml (CLSI/NCCLS, 2003; Winn, 2008).

The suspension was applied to Mueller Hinton agar Petri dishes, aided by a sterile swab, moving it in eight directions so that the plate was fully sown. After about 15 min, the disks impregnated with extracts were applied with the aid of flamed forceps. Then, Petri dishes were inverted and incubated in an oven at 36 ± 1°C for a period of between 16 and 18 h (CLSI/NCCLS, 2003; Winn, 2008).

After the incubation period, the antimicrobial action was read and assessed by the formation of a growth inhibition halo, which was measured using a millimeter rule (CLSI/NCCLS, 2003).

### Statistical Analysis

The tests were conducted in triplicate, and data expressed as mean ± standard deviation (SD). The results were submitted to analysis of variance (ANOVA), the comparison between averages established by Tukey’s HSD *post hoc* test, adopting the 95% level of significance. All analyses were performed with StatSoft software STATISTICS® version 13.3 (Tulsa, United States).

## Results and Discussion

### Efficiency in the Extraction of Flavonoids: Preliminary Tests

The 16 results obtained from the preliminary tests are presented in Table 1 as well as conventional extraction and screening with IL and ES. Most of the tested solvents showed equivalent or superior efficacy, in terms of flavonoid concentration, compared to hydroalcoholic extracts used to relate the alternative solvents’ extraction power with the conventional method. The extract containing [C_6_mim]Cl/water in a 10:1 (g/g) ratio demonstrated twice the extractive potential of 95% ethanol. In contrast, ES CH-BUT and CH-LEV and IL [C_4_mim]Cl with water in the proportion 10:3 (g/g) were those with the lowest efficiency in extract flavonoids from red propolis; however, changing the ratio to 1:1 (g/g) of IL/water, the extraction efficiency doubled, equivalent to the yield obtained with 70% ethanol. In the literature, no studies of propolis extracts with IL were found, but comparing [C_4_mim]Cl extractor potential of flavonoids from heather, it showed superior performance compared to the 60% ethanol extract (Drozdz and Pyrzynska, 2019). The anion of IL acts in disrupting the structure of the natural products’ matrix. At the same time, the imidazolium cation plays an essential role due to the aromatic π-cloud, which strongly interacts with polar and aromatic analytes (Ventura et al., 2017).

**Table 1:** Determination of total flavonoids in red propolis extracts made with different ionic liquids and eutectic solvents, compared with the conventional solvent.

Extractor solvent	Co-solvent	Co-solvent/IL or ES	Total flavonoids (mg RuE. g^−1^)^*^
Ethanol 70%	-	-	112.93^ab^ ± 9.20
Ethanol 95%	-	-	275.85^fgh^ ± 26.91
[C_4_mim]Cl	Ethanol	1:4	290.77^gh^ ± 39.83
[C_6_mim]Cl	Ethanol	1:4	249.48^fgh^ ± 11.36
[C_4_mim][BF_4_]	Ethanol	1:4	229.57^efg^ ± 13.15
[C_4_mim][PF_6_]	Ethanol	1:4	212.05^def^ ± 24.09
[C_4_mim]Cl	Ethanol	1:1	166.11^bcde^ ± 14.56
[C_6_mim]Cl	Ethanol	1:1	298.03^h^ ± 36.17
[C_4_mim][BF_4_]	Ethanol	1:1	153.57^bcd^ ± 7.49
[C_4_mim]Cl	Water	10:3	69.46^a^ ± 14.21
[C_4_mim]Cl	Water	1:1	144.54^bc^ ± 22.98
[C_6_mim]Cl	Water	1:1	179.47^cde^ ± 29.04
[C_6_mim]Cl	Water	10:1	581.06^i^ ± 20.97
CH–BUT	Water	10:1	66.58^a^ ± 8.38
CH–LEV	Water	10:1	62.78^a^ ± 7.95
CH–GLY	Water	10:1	152.97^bcd^ ± 11.90

* Values are equal to mean ± standard deviation (SD). Means followed by the same letter in column do not differ by Tukey’s test at 5% significance. Results expressed in mg of rutin per gram of sample.

Generally, extracts presented different characteristics, according to the solvent and co-solvent used, especially regarding color. The yield of flavonoids of most extracts with the solvents tested in this study showed higher values than conventional extracts found in the literature (Santana Andrade et al., 2017; Devequi-Nunes et al., 2018). The substitution of VOS by alternative solvents (non-volatile solvents) is a promising strategy increasingly addressed. While these alternatives can positively contribute to the environment, there may also be an improvement in terms of the yield of bioactive compounds. Some studies indicate the effectiveness of the use of some ES and IL in the extraction of flavonoids from several natural products, considering conventional solvents. However, no reports compare these solvents in samples of Brazilian red propolis, which have stood out for their biological activities (Koutsoukos et al., 2019; Trusheva et al., 2019).

From the preliminary tests, variables were selected for the experimental design (CCDR 2^3^), analytical viability was considered, associated with the efficiency to extract flavonoids. The IL [C_4_mim][PF_6_] possesses some properties, such as low water solubility, which can cause some difficulties to dissolve with the tested co-solvents. On the other hand, [C_4_mim][BF_4_] is more soluble with the co-solvent; however, this IL’s extraction produced a heterogeneous extract.

Notably, propolis presents a natural resin with a complex composition. Thus, there is an inherent difficulty in standardizing the method for extract preparation with alternative solvents, which also have a high viscosity. In this study, all operational conditions were standardized and further optimized. However, some extracts presented different behaviors, forming multiphase systems, in addition to the formation of an emulsion, making them unfeasible for analysis, such as the case with extracts composed of [C_4_mim][PF_6_] and [C_4_mim][BF_4_], using water as a co-solvent. Those differences highlight the importance of evaluating different solvents and conditions to find the best manner to extract bioactive compounds. Those distinct characteristics may have occurred for fluoridated IL are unstable compounds in water since they hydrolyze, forming by-products that can produce specific toxicity (Plechkova and Seddon, 2008; Freire et al., 2010). For this reason, IL [C_4_mim][PF_6_] and [C_4_mim][BF_4_] were discarded from the screening of solvents. The extract with [C_6_mim]Cl in its largest proportion obtained an increase in flavonoids, about three times concerning the ratio 1:1 (with water).

Among the ES tested, extraction with choline chloride and glycerol did not significantly different compared to the extract with 70% ethanol in flavonoid concentrations. Since alcohol is used as the most common extractor among propolis extracts by several authors (Alencar et al., 2007; da Silva Frozza et al., 2013; Santana Andrade et al., 2017), equivalence in the extractive power of ES is crucial to obtain the exact yield using an alternative solvent. Trusheva et al. (2019) tested the extract with choline chloride and glycerol, and it showed 8.4% of total flavones and flavonols compared to 70% ethanol (11.3%) in propolis samples of poplar type. Funari et al. (2019) tested the same ES, resulting in a lower performance of phenolic compounds than 70% ethanol in green propolis samples. Some authors highlight the influence of water on the ES’s composition, showing that this may be the main factor that induces selectivity in extraction (Liu et al., 2018). The presence of water decreases the viscosity of ES, while it increases their polarity, interfering in the extractive power with low polarity (Funari et al., 2019). However, eutectic mixtures containing more than 30% water must be avoided, as this percentage results in the loss of existing hydrogen bonds, resulting in the de-characterization of the ES structure, which negatively influences its interaction with the flavonoid (Meng et al., 2018). Propolis is mainly composed of flavonoid aglycones and their derivatives, which are predominantly less polar than those glycosylated ones (Zhang et al., 2012; Huang et al., 2014). Thus, a higher percentage of water in ES would reduce the extractive potential of these compounds. On the other hand, ES presents high viscosity, reducing extraction efficiency by slow mass transfer (Fraige et al., 2019). This explains the fact that ES is not effective in extracting propolis flavonoids.

A global evaluation was executed to optimize the extraction methods using alternative solvents and, although the IL [C_4_mim]Cl has formed a homogeneous extract, with a high capacity to extract flavonoids, [C_6_mim]Cl performed better with water as a co-solvent, being this solvent chosen for the stage of extraction optimization, through the experimental design using response surface methodology (RSM).

### Optimization of Ionic Liquid Extraction

#### Central Composition Design Rotational

As mentioned before, [C_6_mim]Cl showed better extraction yield in total flavonoids based on the preliminary test, and it was selected for the experimental design using a CCDR 2^3^ (Table 2). Two responses were considered as results from the assays performed using the conditions specified by the experimental design, flavonoid yield, and antioxidant activity. All results are presented in Table 2.

**Table 2:** Design of experiment 2^3^ using 1-butyl-3-methylimidazolium chloride [C_6_mim][Cl] and 10% water as a co-solvent.

Test	Number of extractions (*X*_1_)	Time of extraction (min; *X*_2_)	Cavitation power (W; *X*_3_)	Flavonoids (mg RuE. g^−1^)^*^	ORAC (μmol TE. g^−1^)	Predictive ORAC (μmol TE. g^−1^)	Deviation (%)
1	3 (−1)	5.0 (−1)	200 (−1)	398.72	4173.39	5163.56	−23.73
2	5 (+1)	5.0 (−1)	200 (−1)	386.55	6538.71	7398.45	−13.15
3	3 (−1)	10.0 (+1)	200 (−1)	260.85	4678.07	5163.56	−10.38
4	5 (+1)	10.0 (+1)	200 (−1)	392.82	7012.41	7398.45	−5.51
5	3 (−1)	5.0 (−1)	400 (+1)	376.65	5596.88	5163.56	7.74
6	5 (+1)	5.0 (−1	400 (+1)	323.09	8034.37	7398.45	7.92
7	3 (−1)	10.0 (+1)	400 (+1)	355.54	4244.95	5163.56	−21.64
8	5 (+1)	10.0 (+1)	400 (+1)	307.04	8033.95	7398.45	7.91
9	2 (−1.68)	7.5 (0)	300 (0)	303.48	3921.17	3453.96	11.91
10	6 (+1.68)	7.5 (0)	300 (0)	384.51	6493.30	7208.58	−11.02
11	4 (0)	3.3 (−1.68)	300 (0)	470.96	8689.50	8011.80	7.80
12	4 (0)	11.7 (+1.68)	300 (0)	383.30	8019.79	8011.80	0.10
13	4 (0)	7.5 (0)	130 (−1,68)	447.60	5290.34	5331.27	−0.77
14	4 (0)	7.5 (0)	470 (+1.68)	432.01	6480.77	5331.27	17.74
15	4 (0)	7.5 (0)	300 (0)	488.26	5794.64	5331.27	8.00
16	4 (0)	7.5 (0)	300 (0)	435.65	6621.68	5331.27	19.49
17	4 (0)	7.5 (0)	300 (0)	428.47	3966.62	5331.27	−34.40

The numbers in parentheses correspond to the coded values in the CCDR.

* The model generated for this response was not predictive.

The flavonoid yield values obtained ranged between 260.85 and 470.96 mg RuE. g^−1^ of the sample. For the CCDR, three variables were considered: the number of extractions (*X*_1_), time of extraction (min; *X*_2_), and cavitation power (W; *X*_3_); however, after data evaluation using 95% of confidence, the effect of variables assessed was not statistically significant on the evaluated response. Also, no significant interaction between the variables used (*R*^2^ < 0.7) was observed. Therefore, data could not be adjusted, and it was not possible to generate a predictive model as well as an equation representing the conditions evaluated. It is difficult to indicate a reason for the lack of adjusting among the experimental data and the predictive values. Often this occurs due to several factors acting together.

Regarding the antioxidant activity against peroxyl radicals, the CCDR assays provided values ranging between 3921.17 and 8689.50 μmol TE. g^−1^ of extract, and two variables were considered to obtain Equation 1: number of extractions (*X*_1_) and time of extraction (*X*_2_). The equation is constructed considering only terms representing the variables with significative effects over the response (antioxidant activity). Thus, not statistically significant terms were incorporated into the lack of fit to calculate the *R*^2^ and *F* ratio.

$$\begin{array}{l} Antioxidantactivity\;\left(\mu \mathrm{mol}\;\mathrm{TE}.g^{-1}\right)=5331.3+117.4\;\left(X_{1}\right)+\\ \;\;\;\;\;\;\;\;\;\;\;\;\;\;\;\;\;\;\;\;\;\;\;\;\;\;\;\;\;\;\;\;\;\;\;\;\;\;\;\;\;\;\;\;\;\;\;\;\;\;\;\;\;\;\;\;\;\;\;\;\;\;\;\;\;\;\;\;\;\;\;\;\;\;\;\;\;\;\;\;\;\;\;\;\;\;\;\;\;\;\;\;\;\;\;\;\;\;\;949.7{\left(X_{2}\right)}^{2} \end{array}$$ (1)

The conditions provided the best antioxidant activity (8689.50 μmol TE. g^−1^ of the sample) from experiment 11 (Table 2), whose conditions were: four extractions with the same biomass, time of extraction at 3.3 min, and 300 W of cavitation power. The dependent (antioxidant activity) and independent variables were fitted to a second-order model, and they were examined in terms of goodness of fit. Therefore, ANOVA was used to evaluate the adequacy of the fitted model.

**Figure 1:**
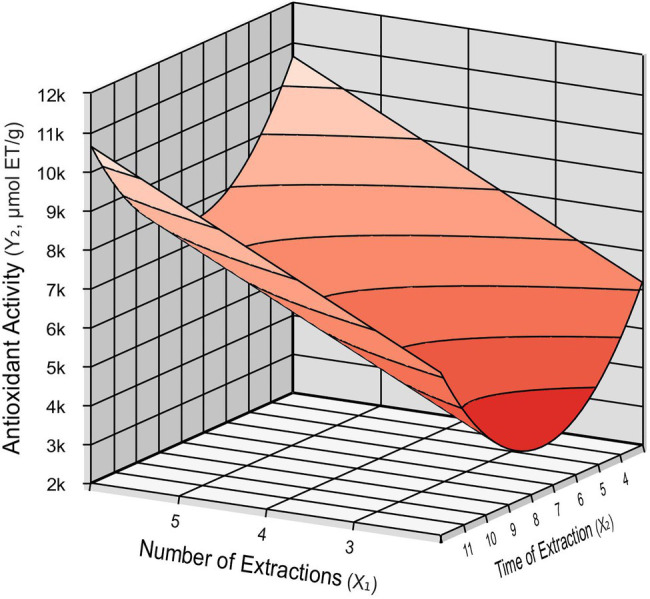
Response surface for antioxidant activity as a function of the number of extractions (*X*_1_) and time of extraction (*X*_2_).

In this study, *R*^2^ was 0.70, and the estimated *F* value was approximately five times the *F* tabulated for antioxidant activity, showing that the proposed model was predictive, with a 95% confidence level. The obtained model for antioxidant activity was used to construct the response surface presented in Figure 1 to understand the interactions between the number of extractions (*X*_1_) and time of extraction (*X*_2_) required to maximize the antioxidant activity provided by the red propolis extracts.

Analyzing the response surface and the contour diagram presented in Figure 1, two work regions provide the optimal conditions for antioxidant activity, evaluating the number of extractions and time of extractions. Both areas are comprising a higher number of extractions. The minimum and maximum levels can be considered for variable extraction time since both presented high antioxidant activity values in propolis extracts. The results show that [C_6_mim]Cl has a strong extraction potential, intensified with each repetition of the process, even at the lowest extraction time tested, exhibiting affinity with the bioactive compounds contained in propolis, which represents an advantage in the extraction process, confirmed by the better performance in antioxidant activity assay.

Despite no model being obtained for flavonoids concentrations, the highest value obtained, considering the assays based on the CCDR, for the flavonoid yield was provided by the same conditions presented in the predictive model for antioxidant activity, showing a possible relationship between the flavonoids contained in red propolis in their antioxidant action.

### Experimental Validation

Based on the response surface analysis (Figure 1), we determined the extraction condition in which higher antioxidant activity is obtained. Such conditions are shown in Table 3, which indicates separately the values in which variables *X*_1_, *X*_2_, and *X*_3_ provided the best values for antioxidant activity from the red propolis extracts.

**Table 3:** Extraction condition for the antioxidant activity model validation.

Independent variables	Levels
Number of extractions (*X*_1_)	0 (4)
Time of extraction (*X*_2_)	−1.68 (3.3 min)
Cavitation power (*X*_3_)	0 (300 W)

The validation was carried out employing an experimental test under these conditions, presenting, by determining the extract antioxidant activity, 7595.77 μmol TE. g^−1^, a deviation of 5.48% concerning the result predicted by the experimental design. The results obtained confirm that the obtained model is predictive and reproducible for antioxidant activity, considering the assessed conditions in this work. Results presented related to antioxidant activity were superior to those obtained by some authors, such as Santana Andrade et al. (2017, 6,665 μmol TE. g^−1^). This optimized extract showed 394.39 ± 36.30 mg RuE. g^−1^ regarding total flavonoids, highlighting the close relationship between antioxidant activity and flavonoid concentration in the propolis extract, which has high levels of these polyphenols.

In the commercial context, the process of obtaining alcohol-free propolis extract is notably relevant. In countries, where most of the population is Muslim, less consumption of alcohol occurs, since it is forbidden to drink alcoholic beverages in the Islamic religion, requiring control in the production and import of alcohol in these countries (Al-ansari et al., 2015, 2019; Amin-Esmaeili et al., 2018). In addition to this group, abstainers and children are others who would be restricted from consuming alcoholic extracts. Although today various options for aqueous propolis extracts exist and studies related to them, hydroalcoholic extracts, in many cases, perform better in terms of biological activities (Hazem et al., 2017; Sayyadi et al., 2020). The proposal presented in this work represents an advantage concerning other processes in the yield of bioactive compounds.

### Red Propolis Extract Flavonoid Composition

**Figure 2:**
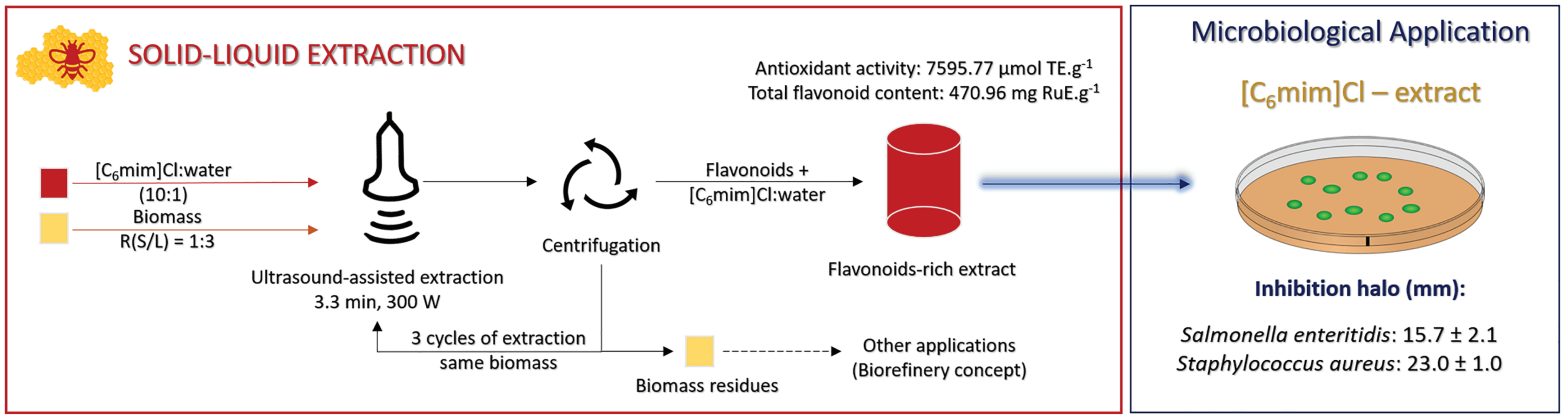
Optimized process designed for high recovery of phytochemicals from red propolis.

**Figure 3:**
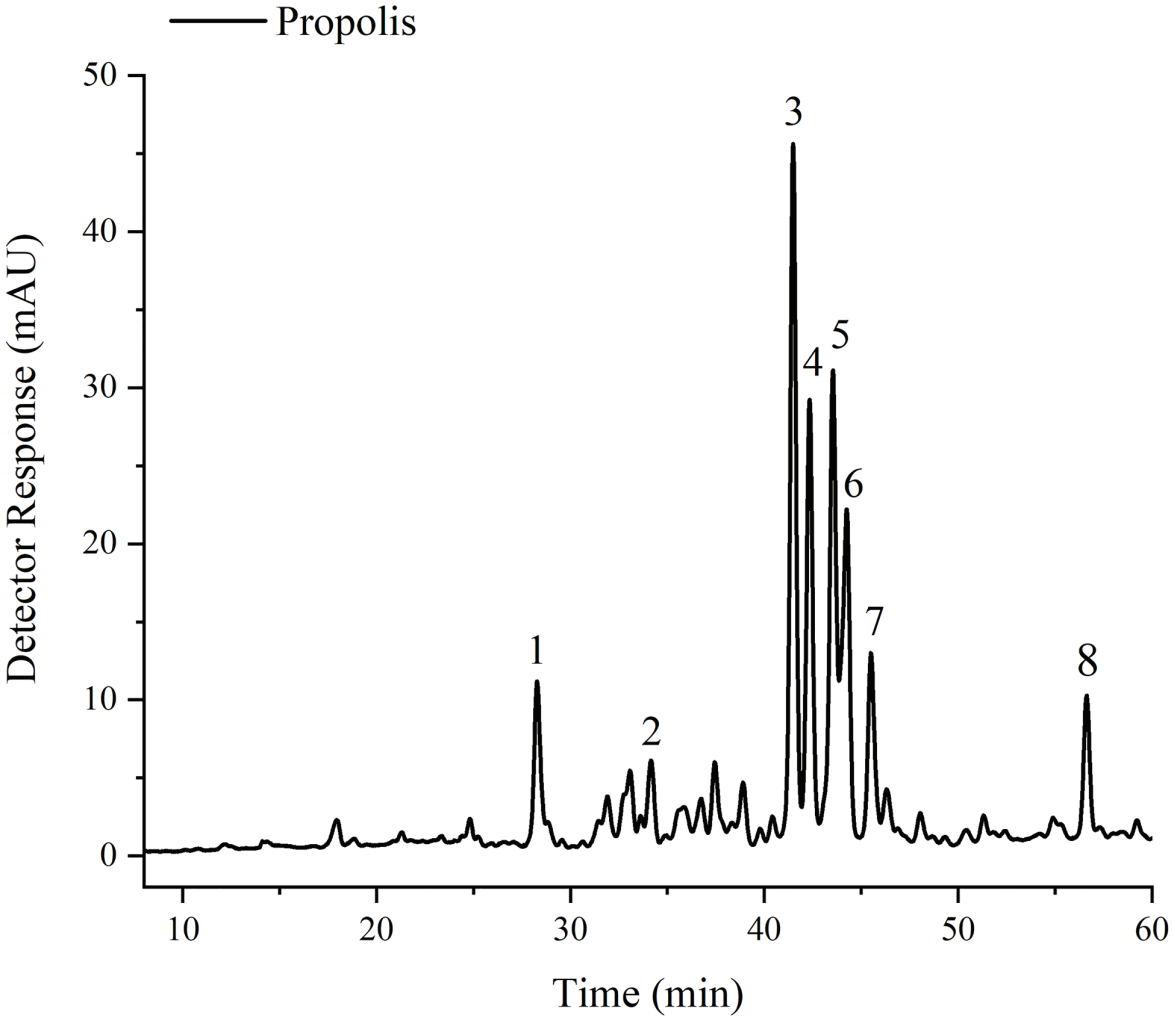
Typical chromatogram of red propolis flavonoids extract obtained from [C_6_mim]Cl. Chromatograms were processed at 280 nm.

The optimized and validated extracts were analyzed by high performance liquid chromatography (HPLC; Figure 2) and chromatogram, as shown in Figure 3. The characteristics of the flavonoids separated in red propolis extract are shown in Table 4. Medicarpin (peak 3) and isoliquiritigenin (peak 5) were the major constituents in an extract with [C_6_mim]Cl, representing 24 and 18% of total flavonoids content, respectively, followed by hesperetin derivative (peak 4) and formononetin (peak 6), which contributed 17 and 12%, respectively. The extraction processes using hydrophobic IL [C_6_mim]Cl proved to be selective for more hydrophobic flavonoids (medicarpin). Thus, the higher hydrophobicity of the IL implies the extraction of the most hydrophobic components of the biomass because of the π–π, n–π, and hydrophobic bonds (de Souza Mesquita et al., 2019; Murador et al., 2019b).

**Table 4:** Chromatographic and UV-visible (UV-vis) characteristics of the flavonoids identified in the red propolis extract obtained from [C_6_mim]Cl.

Peak	*t_r_* (min)^a^	Compound^b^	*λ*_max_ (nm)^c^,^d^	Standard Co-chromatography	References
1	27.5	Liquiritigenin	205, 246, 276, 312	No	Li et al., 2015
2	33.3	Daidzein	238, 249, 259 (sh), 303 (sh)	Yes	Marques das Neves et al., 2016
3	40.6	Medicarpin	237, 242, 281	No	da Silva Frozza et al., 2013
4	41.5	Hesperetin derivative	237, 242, 284	No	da Silva Frozza et al., 2013
5	42.6	Isoliquiritigenin	280, 370	No	Li et al., 2015
6	43.4	Formononetin	215, 244, 305	Yes	Srivastava et al., 2015; Marques das Neves et al., 2016
7	44.6	Biochanin A	260, 310 (sh)	No	do Nascimento et al., 2016
8	55.8	Pinocembrin	244, 280	No	do Nascimento et al., 2016

a Retention time on the C18 column.

b Tentatively identified.

c Linear gradient of methanol/acidified water.

d *λ*_max_: maximum absorption wavelength (nm).

Red propolis can be found in tropical countries like Brazil, Cuba, Venezuela, and Mexico (Alencar et al., 2007; Cuesta-Rubio et al., 2007; Lotti et al., 2010; López et al., 2014). In research on the phytochemical markers of red propolis, daidzein was not found in all samples from Alagoas, the same geographical origin as the sample in this study. At the same time, formononetin and biochanin A were found in all studied samples (López et al., 2014).

This result demonstrates the variety in propolis chemical composition, although some authors have found these compounds in samples of red propolis. It is known that propolis chemical composition varies according to original geographic region, and propolis phytochemical profile can vary due to several factors such as local flora, season, and extraction methods, resulting in different extracts (Alencar et al., 2007; Cavalcanti de Pontes et al., 2018; do Nascimento et al., 2019). da Silva Frozza et al. (2017) identified some compounds of biological interest, such as isoflavones formononetin, and biochanin A besides isoliquiritigenin in samples of red propolis from Alagoas. These compounds were found in this study, but others flavonoids demonstrating once again the variability of this type of extract (Bankova, 2005; Escriche and Juan-Borrás, 2018; do Nascimento et al., 2019).

#### Antimicrobial Activity Test

The extract containing IL presented antimicrobial activity against the two bacteria tested, as described in Table 5. The optimized extract pure with IL (394.39 mg RuE. g^−1^) and the negative control with IL [C_6_mim]Cl presented no significant difference in the antimicrobial action against both tested bacteria, indicating that this solvent contributed to the antimicrobial effect. Some review articles report the antimicrobial activity of imidazolium-based IL (Reddy and Nancharaiah, 2020; Sivapragasam et al., 2020). The mechanism of IL antimicrobial activity is not known. Still, there is evidence that the long alkyl chain linear of the cation positively influences this activity, which may disrupt membrane proteins (Docherty and Kulpa., 2005; Duman et al., 2019; Florio et al., 2019).

**TableT 5:** Antibiogram of red propolis extracts.

Inhibition halo (mm)^*^		
Tested extract^**^	*Salmonella enteritidis*	*Staphylococcus aureus*
C(+)^***^	34.7^d^ ± 1.5	20.4^b^ ± 0.5
NC N.d.	15.0^b^ ± 0.0	22.0^bc^ ± 0.0
NC25	0.0^a^ ± 0.0	7.2^a^ ± 0.3
NC75	0.0^a^ ± 0.0	8.2^a^ ± 1.0
EXT N.d.	15.7^b^ ± 2.1	23.0^c^ ± 1.0
EXT25	0.0^a^ ± 0.0	8.0^a^ ± 0.9
EXT75	6.8^c^ ± 0.8	10.3^d^ ± 0.6

* Values are mean ± standard deviation (SD). Means followed by the same letter in column do not differ by Tukey’s test at 5% significance.

** C(+) = Positive control; NC N.d. = Negative control no dilution; NC25 = Negative control 25 mg ml^−1^; NC75 = Negative control 75 mg ml^−1^; EXT N.d. = Extract no dilution (394 mg RuE. g^−1^); EXT25 = Extract 25 mg.ml^−1^ (7,9 μg RuE/disc); EXT75 = Extract 75 mg.ml^−1^ (23,8 μg RuE/disc).

*** Meropenem (10 μg/disc) – *Salmonella enteritidis* and Vancomycin (30 μg/disc) – *Staphylococcus aureus*.

The optimized process is presented in Figure 2. The disc with the extract in the concentration of 75 mg ml^−1^, which contains 23.8 μg RuE/disc, showed antimicrobial action against *Sa. enteritidis*. The extract in the concentration of 25 mg ml^−1^ (7,9 μg RuE/disc) presented no antimicrobial activity for these bacteria. There were no comparative studies in the literature regarding the effect of the red propolis against this bacterium. Still, it is known that Gram-negative bacteria tend to better resist these compounds due to the intense lipid layer, making the cell wall impermeable for several macromolecules (Efenberger-Szmechtyk et al., 2021). Despite this, with approximately twice the extract concerning the amount of antibiotics, it was possible to verify antimicrobial activity against this Gram-negative bacterium.

The antimicrobial action against *St. aureus* from the extract with IL (394 mg RuE. g^−1^) was statistically higher concerning the positive control for this bacterium. The extracts in all dilutions showed antimicrobial activity against this Gram-positive bacterium, including negative control. Despite this, the extract diluted to a concentration of 75 mg. 10^−1^ showed more significant inhibition compared to IL in the same conditions.

The antimicrobial action of the red propolis extract in Gram-positive bacteria has already been evidenced by some authors, who have also found promising results on the activity against the bacterium *St. aureus* (Souza Machado et al., 2016; Dantas Silva et al., 2017; Regueira-Neto et al., 2018; Silva et al., 2019).

The excellent performance of the red propolis extract with [C_6_mim]Cl can be attributed to the increase in phytochemicals achieved by the extraction. At the same time, regarding negative control, the IL, as it is non-volatile, remained impregnated in the disc. Thus, tests involving other microbiological techniques are necessary to compare with the results obtained. Besides, methods for separating the IL from the extract – which is one of the advantages of using IL, as it can be recycled and reused – must be carried out to enhance the extract viability and to minimize possible toxic effects originated at the IL (do Nascimento et al., 2019).

The determination of toxicity and antimicrobial activity is crucial to release an IL to the industry. Although these compounds are considered sustainable due to their possibility of being recycled and reused, non-flammability and non-volatility are characteristics that associate them with “green” chemistry. Some articles have shown some IL based on imidazolium that are associated with toxicity. Therefore, it was impossible to consider IL as genuinely green compounds (Plechkova and Seddon, 2008). Cationic elements of IL play an essential role in microorganisms, depending on the length of the alkyl chain, contributing to toxicity (Fister et al., 2017) with significant antimicrobial activity against Gram-positive and Gram-negative bacteria (Docherty and Kulpa., 2005; Patil et al., 2020; Sivapragasam et al., 2020). This may explain the action of the IL against the tested bacteria. Thus, it is necessary to understand the behavior of both IL and red propolis individually to verify if, in fact, the sample showed antimicrobial activity or if the effect shown was overestimated by the action of the IL on bacteria.

On the other hand, the antimicrobial activity of [C_6_mim]Cl can be an advantage to enable the use of the extract in several applications, such as food packaging incorporated into biodegradable films (de Souza Mesquita et al., 2020a,b). The optimized extracts supported the antimicrobial activity, inhibiting the growth of both *St. aureus* and *Sa. enteritidis*, the latter being a Gram-negative bacterium, a type known to obtain a more complex cell wall, rich in lipopolysaccharides. An interesting point was the antimicrobial action of pure [C_6_mim]Cl, suggesting that this solvent interacts with the cellular components of the bacterial wall, generating the need to know these mechanisms, in addition to also evaluating the toxicity in human cells.

## Conclusion

Propolis is known for thousands of years for its diverse biological actions. However, its use is limited in certain groups. The study of alternative solvents, moreover, to be an appealing commercial strategy from an economic point of view, enables expanded research on propolis chemical composition. [C_6_mim]Cl was able to extract total flavonoid contents better than ethanol in the two concentrations tested in the screening tests. Furthermore, the optimization of the process determined the best conditions for preparing highly antioxidant extracts. Thus, it was possible to obtain a high-performance extract regarding flavonoids, with antioxidant and antimicrobial activity, as to expose in Figure 3, we developed a complete and optimized process designed for high recovery of phytochemicals from red propolis, compared to the methods previously developed in the literature mediated by VOS or ES.

In summary, we developed an ultrasound-assisted extraction under the operational conditions optimized at three cycles of extractions with the same biomass, up to 3.3 min at 300 W of cavitation, which was possible to recover 470.96 mg of phytochemicals with high antioxidant activity (7595.77 μmol TE. g^−1^). Besides, the biomass residues can be used for other applications, applying the biorefinery concepts, which are extremely important in a sustainable society. In the end, the extracts were evaluated by microbiological assays, showing that the use of IL displays positive effects against *Sa. enteritidis* and *St. aureus*.

Nevertheless, some steps are still necessary in order to promote the characterization of this complex biomass and spur future investigations to minimize the effects inherent to the process, including the recycling and reuse of the [C_6_mim]Cl, to make the method more sustainable. Besides, we suggest that future studies focus on understanding IL’s interaction with the phytochemicals extracted from red propolis as a way to use it more effectively, better using these benefits from its rich composition in phytochemicals, also assuring the sustainable use of the raw material.

## Data Availability Statement

The raw data supporting the conclusions of this article will be made available by the authors, without undue reservation.

## Author Contributions

CS contributed to the execution and analysis of all experiments. VR contributed to the planning of experiments. AB contributed to the analysis and interpretation of the data included. All authors wrote and reviewed the results and approved the final version of the manuscript.

### Conflict of Interest

The authors declare that the research was conducted in the absence of any commercial or financial relationships that could be construed as a potential conflict of interest.
